# Role of long non-coding RNA *H19* in the development of osteoporosis

**DOI:** 10.1186/s10020-021-00386-0

**Published:** 2021-09-28

**Authors:** Senxiang Chen, Da Liu, Zimo Zhou, Sen Qin

**Affiliations:** Department of Orthopedics, Shengjing Hospital of China Medical University, Shenyang, 110004 Liaoning China

**Keywords:** Long noncoding RNA, lncRNA *H19*, Osteogenesis, Osteoporosis

## Abstract

**Background:**

Osteoporosis is a widespread and serious metabolic bone disease. At present, revealing the molecular mechanisms of osteoporosis and developing effective prevention and treatment methods are of great significance to health worldwide. LncRNA is a non-coding RNA peptide chain with more than 200 nucleotides. Researchers have identified many lncRNAs implicated in the development of diseases and lncRNA *H19* is an example.

**Results:**

A large amount of evidence supports the fact that long non-coding RNA (lncRNA) genes, such as *H19*, have multiple, far-reaching effects on various biological functions. It has been found that lncRNA *H19* has a role in the regulation of different types of cells in the body including the osteoblasts, osteocytes, and osteoclasts found in bones. Therefore, it can be postulated that lncRNA *H19* affects the incidence and development of osteoporosis.

**Conclusion:**

The prospect of targeting lncRNA *H19* in the treatment of osteoporosis is promising because of the effects that lncRNA *H19* has on the process of osteogenic differentiation. In this review, we summarize the molecular pathways and mechanisms of lncRNA *H19* in the pathogenesis of osteoporosis and summarize the research progress of targeting *H19* as a treatment option. Research is emerging that explores more effective treatment possibilities for bone metabolism diseases using molecular targets.

## Background

Osteoporosis is a widespread metabolic bone disease and the risk of developing it increases with age (Zou et al. 2020). The main characteristics of this debilitating disease are bone loss, bone density reduction, bone fragility, and the risk of fractures (Rachner et al. 2011). Because of problems with underdiagnosis, it is difficult to determine the variation in the incidence and prevalence of osteoporosis worldwide. Therefore, the best way to compare osteoporosis among different population groups is to observe the fracture rates (Johnston and Dagar 2020). Worldwide, osteoporosis causes more than 8.9 million fractures annually, resulting in an osteoporotic fracture every 3 s (Noh et al. 2020). As life expectancy is increasing globally, osteoporosis will cause a severe economic burden in most countries. Osteoporosis-related fractures cost approximately $17.9 and £4 billion per annum in the USA and the UK, respectively (Hernlund et al. 2013). And it is estimated that the worldwide economic cost of osteoporosis is expected to increase to $131.5 billion by 2050 (Harvey et al. 2010).

The occurrence of osteoporosis is related to many genetic and environmental factors. These factors mainly affect the incidence and development of osteoporosis by regulating the activity and differentiation of osteoblasts and osteoclasts (Trajanoska and Rivadeneira 2019). Human bone metabolism includes bone formation by osteoblasts and bone resorption by osteoclasts. There are existing drugs to treat osteoporosis that inhibit the activity of osteoclasts. These include anti-bone resorption drugs such as bisphosphonates and the receptor activator of nuclear factor-kb ligand (RANKL) inhibitor, denosumab, which inhibits osteoclast formation, function, and survival. These drugs, however, have complicated side effects, and there is currently no optimal drug treatment for osteoporosis (Khan et al. 2017). Consequently, the exploration of targets for anabolic therapy, or the stimulation of bone formation by pharmacologic means, is an important emerging research focus. Understanding the molecular mechanisms of osteoporosis and developing effective prevention and treatment methods targeted to these mechanisms are of great importance to the health of an aging world population.

Recently, epigenetic studies have provided new insights into the treatment of osteoporosis. Epigenetic changes affect gene expression without changing the DNA sequence (Yang et al. 2020) and include DNA methylation, histone modification, and RNA-based mechanisms (van Wijnen and Westendorf 2019). With recent research developments, accumulating data suggest that lncRNA plays an important role in regulating bone cells and, therefore, affects the occurrence and development of osteoporosis (Hassan et al. 2015).

LncRNA is a non-coding RNA peptide chain with more than 200 bases and does not translate protein. Previously regarded as translation noise, lncRNA is now a prime focus of study in the rapidly developing field of genomics (Schmitz et al. 2016). Researchers have identified many lncRNAs implicated in the development of diseases and lncRNA *H19* is an example. For instance, lncRNA *H19* regulates multiple myeloma by targeting *miRNA-152-3p* (Zheng et al. 2020) and is also a therapeutic target for pancreatic cancer (Sun et al. 2019). However, the process of how lncRNA *H19* modulates the pathogenesis of osteoporosis remains unclear. In this review, we discuss the role of lncRNA *H19* in osteogenic differentiation, and summarize the relevant mechanisms of action to provide a theoretical basis for exploring lncRNA as a new molecular target in the treatment of osteoporosis.

## Discussion

### Sources and classification of lncRNAs

With the advancement of gene sequencing technology, a large number of non-coding lncRNAs have been discovered. LncRNA is a highly structured RNA transcription product. However, owing to the lack of an open reading frame, it does not translate protein and directly regulates gene expression at the RNA level (Jathar et al. 2017). It is also widely present in the cytoplasm and nucleus after transcription (Ponting et al. 2009). Generally, lncRNAs do not have coding potential, but some lncRNAs can code short peptides (Rion and Rüegg 2017). LncRNA is mostly transcribed by RNA polymerase II and most lncRNA transcripts have 5′-methylated caps and 3′-polyadenylic acid tails (Wu et al. 2013). However, some lncRNAs are cleaved by the microprocessor to terminate transcription, or are bidirectionally transcribed, resulting in lncRNAs without 5′-methylated caps and 3′-end poly tails (Chen 2016).

LncRNAs can originate in various ways (Ponting et al. 2009) such as, by the disruption of the translation reading frame of protein-coding genes, from chromosomal reorganization, by non-coding genes through reverse transcription, and by the generation of non-coding RNA containing adjacent repeats through a partial tandem replication mechanism (Wei et al. 2017). LncRNA is highly involved in a variety of biological functions, but compared to the research of protein-coding sequences and micro-RNA (miRNA), lncRNA research is still early in its development. Extensive studies have been conducted on miRNA, and researchers have found that it can completely or incompletely base pair with target genes to regulate gene expression (Pu et al. 2019). In comparison, lncRNA has a longer molecular sequence, a spatial structure, and a variety of modes of action, all of which allow for the possibility of many exploratory research pathways (Krol et al. 2015). The secondary structure, splicing form, and subcellular location of most lncRNAs are relatively conservative. This is particularly important for lncRNAs to function (Kopp and Mendell 2018). LncRNA can be divided into five categories according to their positions and genome sequences: sense, antisense, bidirectional, intronic, and intergenic (Ma et al. 2013; Wu et al. 2018a, b). In addition, lncRNAs can be divided into bait molecules, signal molecules, primers, and framework molecules according to molecular mechanisms (Wang and Chang 2011). These different types of lncRNAs perform a wide variety of functions.

### Common functions of lncRNAs

LncRNAs have time and space specificity. The expression of lncRNAs varies in different tissues and the expression of lncRNA within the same tissue can also differ (Kim and Sung 2012). Because of these characteristics of lncRNA, its mechanism of action is complicated. Presently, a measure of the extent of all the actions of lncRNAs has not been fully elucidated, but there are roughly four types according to current research (Peng et al. 2018).

One type of lncRNA mechanism of action involves the regulation of epigenetic modification. Certain lncRNAs recruit chromatin remodeling and modification complexes to specific sites (Fang and Fullwood 2016). This changes the DNA/RNA methylation status, chromatin structure, and modification status. As a result, downstream gene expression is regulated by mediating chromatin remodeling or histone modification. For instance, hypomethylation of the promoter region of lncRNA *SOX21-AS1* can make it overexpress, thereby enhancing the inhibitory effect of lncRNA *SOX21* on lncRNA *SOX2* and inhibit the progression of cervical cancer (Wang et al. 2019).

Another function of lncRNA is the regulation of transcriptional expression. In eukaryotic cells, transcription factors are vital for gene transcription. They can bind to the RNA produced by gene transcription to control RNA transcription, localization, and stability. Some lncRNAs act as ligands and bind to transcription factors to form a complex to control gene transcription activity by affecting the promoter region or adjacent genes (Dykes and Emanueli 2017). For instance, lncRNA *GATA3-AS1* is a divergent lncRNA adjacent to lncRNA *GATA3* and the *GATA3-AS1* transcript alone is sufficient to induce an increase in *GATA3* expression (Gibbons et al. 2018).

Dictating post-transcriptional regulation is an additional process in which lncRNA is involved. LncRNA participates in processes such as shearing, editing, protein translation, and transcription after mRNA transcription. By forming complementary double strands with mRNA, it interferes with the shearing of mRNA to produce a regulatory effect (Ransohoff et al. 2018).

The fourth type of lncRNA action is to function as competing endogenous RNA (ceRNA) to bind miRNA like a sponge, thereby preventing miRNA from binding to target mRNA. LncRNA also forms complementary duplexes with mRNA and small interfering RNA (siRNA) to regulate gene expression (Chan and Tay 2018).

### Mechanisms of action of lncRNAs in bone development

Adult bone mass is maintained in homeostasis. To accomplish this, new bone formation replaces old bone tissue so that bone mass maintains a dynamic balance (Zhou et al. 2021). Osteoblasts, osteoclasts, and osteocytes are the main cells involved in this bone remodeling process. Osteoblasts are the main functional cells in the process of bone tissue formation. They are responsible for the synthesis, secretion, and mineralization of the bone matrix and are mainly differentiated from bone mesenchymal stem cells in the periosteum and bone marrow (Chen et al. 2015). Mesenchymal stem cells (MSCs) are pluripotent cells that can differentiate into osteoblasts, adipocytes or chondrocytes, and have become the preferred source of osteoporosis-based cell therapy. A variety of signaling pathways are involved in bone formation to regulate bone homeostasis, such as the Wnt/β-catenin pathway, Notch pathway, and MAPK pathway (Majidinia et al. 2018). After the Wnt/β-catenin pathway is activated, glycogen synthase 3 activity is reduced and β-catenin phosphorylation and proteasome degradation are inhibited. The stabilized β-catenin enters the nucleus to activate gene transcription and induce the proliferation and differentiation of osteoblasts (Nusse 2012). LncRNA regulates bone formation by affecting these signaling pathways. LncRNA *ANCR* affects the Wnt/β-catenin pathway and inhibits osteogenic differentiation (Jia et al. 2015), while lncRNA *HOTAIR* affects the Wnt/β-catenin pathway and inhibits osteogenic differentiation (Shen et al. 2019). The MAPK signaling pathways JNK, ERK 1/2, and p38 play an important role in osteogenic differentiation (Ba et al. 2017). Knockdown of lncRNA *DANCR* can activate p38 MAPK to induce MSCs to differentiate into osteoblasts (Zhang et al. 2018). In addition, lncRNA *SNHG1* can inhibit the activation of p38, thereby inhibiting the osteogenic differentiation of bone marrow mesenchymal stem cells (BMSCs) (Jiang et al. 2019).

**Fig. 1:**
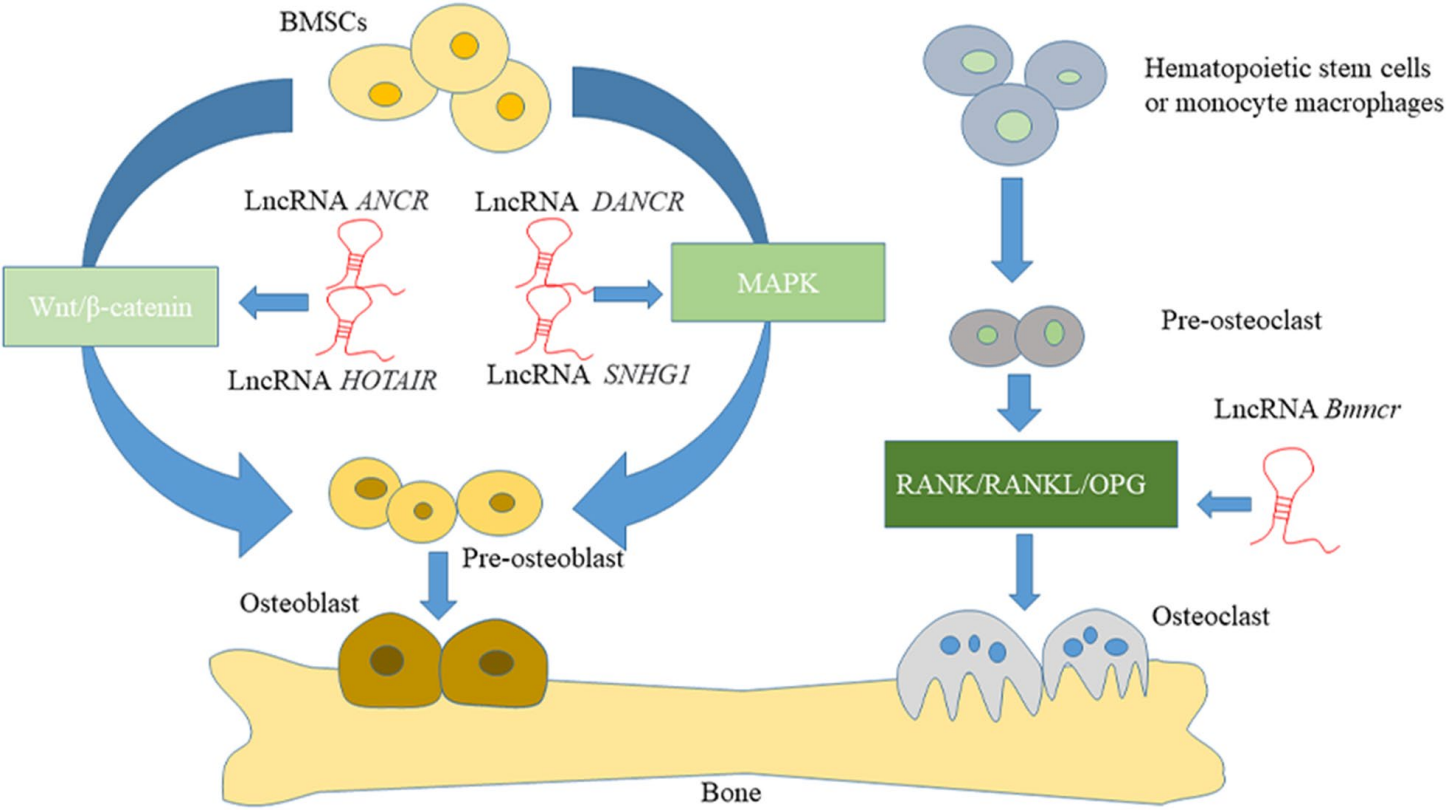
Mechanisms of action of lncRNAs in bone development. LncRNA regulates osteogenic differentiation through Wnt/β-catenin and MAPK pathways. lncRNA *ANCR* and lncRNA *HOTAIR* affect the Wnt/β-catenin pathway and inhibit osteogenic differentiation. LncRNA *DANCR* and LncRNA *SNHG1* regulate osteogenic differentiation through the MAPK pathway. LncRNA regulates osteoclast differentiation through RANK/RANKL/OPG and other pathways. LncRNA *Bmncr* inhibits RANKL-induced osteoclast differentiation

Osteoclasts are derived from hematopoietic stem cells or mononuclear macrophages. They can differentiate into osteoclast precursor cells and mature under the regulation of RANKL secreted by osteoblasts (Ono and Nakashima 2018). RANKL is a key cytokine that regulates the differentiation of osteoclasts. It combines with the signal receptor RANK to promote the formation of osteoclasts. The osteoprotegerin (OPG) secreted by osteoblasts inhibits the combination of RANKL and RANK and inhibits the formation of osteoclasts. RANK/RANKL/OPG is the main regulatory system for osteoclast differentiation, maturation, and survival (Udagawa et al. 2021). When osteoclast activity increases, it causes increased bone resorption and bone destruction (Fig. 1).

There are few studies involving the role of lncRNA in osteoclast differentiation, but some researchers have found that lncRNA does play a role in regulating this process. Chen et al. found that lncRNA bone marrow associated non-coding RNA (Bmncr) could inhibit RANKL-induced osteoclast differentiation and inhibit the progression of osteoporosis (Chen et al. 2019). LncRNA *Jak3* was also found to increase the expression of cathepsin K (CTSK) through Jak3-mediated activation of T-cells 1 (NFATC1). *Jak3* plays a key role in the differentiation of osteoclasts through Jak3/NFATC1/CTSK (Lee et al. 2019). NFATC1 is an essential transcription factor in the process of osteoclastogenesis, and lncRNA *AK077216* promotes the expression of NFATC1 by inhibiting the expression of NFAT interacting protein (NIP45), thereby promoting the differentiation of osteoclasts (Liu et al. 2019).

The functional mechanism of lncRNA allows it to have an important role in the occurrence and development of osteoporosis. For instance, lncRNA *CASC11* is up-regulated in the postmenopausal osteoporosis and is associated with TNF-α (Yu et al. 2019). TNF-α can inhibit the activity of osteoblasts at certain stages of osteogenic differentiation, and stimulate the proliferation and differentiation of osteoclasts (Wang and He 2020). In addition, lncRNA *GAS5* is up-regulated in osteoporosis and may down-regulate *miR-21* to promote osteoclast apoptosis (Cong et al. 2020). Further research is required to elucidate the role of abnormal lncRNA expression in the development mechanism of osteoporosis, as well as to discover the involved pathological effects and molecular mechanisms.

Through the increasing amount of research on this topic, many lncRNAs have been found to regulate important biological activities. Compared with coding RNA and miRNA, the research time involved with lncRNA is not as arduous; there are still many lncRNAs that have not yet been studied, and the function of these lncRNAs is still unclear. Researchers will be able to discover important gene regulatory functions faster and more effectively with the development of more high-throughput screening technologies such as microarray, the combination of a new generation of high-throughput sequencing technology, and using bioinformatics prediction tools. This will allow for more functional mechanisms of lncRNA to be discovered. Significantly, many lncRNAs are involved in bone remodeling and osteogenic differentiation, and they can influence the differentiation and activity of osteoblasts and osteoclasts. Despite the biological evidence, the specific mechanism of this process needs to be better understood through more research.

### LncRNA *H19* in osteoporosis

LncRNA *H19* is located on chromosome 11p15.5 and is 2.3 kb. It is only expressed in maternal alleles and plays an important role in regulating various biological functions, such as cancer and diabetes (Peng et al. 2018). The expression of *H19* is affected by many factors, including oncogenes. *H19* has been confirmed to be up-regulated in many cancers and promotes the proliferation of cancer cells (Ghafouri-Fard et al. 2020). For example, up-regulation of *H19* and *miR-675* promotes the proliferation of gastric cancer cells and inhibits apoptosis, while knocking down these two genes causes the opposite effect (Yan et al. 2017). In colorectal cancer cells, *H19* mediates *miR-138* by sponging it and subsequently enhances the expression of HMGA1, thereby enhancing the invasion ability of cancer cells (Yang et al. 2017). *H19* is one of the most conserved and abundant non-coding transcripts in mammalian development (Gabory et al. 2010) and has been proven to promote bone growth and differentiation (Dey et al. 2014). With the focused research on *H19*, it has been found that *H19* can regulate osteoblast differentiation, mediate bone regeneration, and regulate bone metabolism. Based on the effects of *H19* on the process of osteogenic differentiation, the potential use of *H19* in the treatment of osteoporosis can be studied. In addition, *H19* has many connections to osteoporosis. Li et al. found that the up-regulation of DMNT1 can lead to the methylation of the *H19* promoter in osteoporotic rats and inhibit the ERK signaling pathway, which can cause disuse osteoporosis (Li et al. 2018). Further research is needed into how *H19* regulates osteogenic differentiation and how it participates in osteoporosis.

### Roles of lncRNA *H19* in regulating osteogenic differentiation through modulation of the lncRNA-miRNA-mRNA network

LncRNA *H19* can act as the upstream target gene of miRNAs, thereby regulating the expression of mRNA and influencing the process of osteogenic differentiation of osteoblasts. *H19* is up-regulated during the process of osteogenic differentiation of MSCs, while knockdown of *H19* inhibits the proliferation of osteoblasts, both in vivo and in vitro*.* Huang et al. implanted hMSCs stably expressing *H19*, sh*H19*-1 and control hMSCs into subcutaneous tissues of mice. Eight weeks later, it was observed that the bone formation of the *H19*-overexpressing group was higher than the control group, while the bone formation of the *H19*-knockdown group was reduced, which was consistent with the results of in vitro cell experiments (Huang et al. 2015). Studies have shown that *miR675*, which is inserted into the first exon of *H19*, promotes the osteogenic differentiation of human mesenchymal stem cells. *H19* is the precursor of *miR675*, and its first exon contains a hairpin consisting of miRNA. This hairpin was found to be a template of two different miRNAs (*miR-675-5p* and *miR-675-3p)* that can express *miR-675* with 23 nucleotides (Cai and Cullen 2007; Keniry et al. 2012). In addition, *H19* forms self-regulating feedback with its encoded *miR-675-5p*. This indicates that *miR-675-5p* targets *H19* to form a negative regulatory loop. Both *H19* and *miR675* are up-regulated in osteoporosis differentiation and *miR-675* down-regulates TGF-β1 (Huang et al. 2015). Down-regulation of TGF-β1 inhibits phosphorylation of Smad3 (Derynck and Zhang 2003), and it reduces the expression of histone deacetylase (HDAC4/5) required for osteogenic differentiation. Phosphorylation of Smad3 recruits HDAC4/5 to Runx2 and forms a stable complex on the DNA sequence bound by Runx2, thereby down-regulating the expression of Runx2 (Kang et al. 2005). The *miR675* partially mediates the osteogenic activity of *H19*, so the *H19*/*miR-675*/TGF-β1/Smad3/HDAC pathway can promote osteogenic differentiation, but further studies are needed to determine whether *H19*/*miR-675* directly targets these factors or involves other mechanisms.

Bone morphogenetic proteins (BMPs) belong to the TGF-β superfamily and play an important role in osteogenic differentiation and bone formation. BMP9 is the most significant type of BMPs that induces the osteogenic differentiation of MSCs. Interestingly, recent studies have found that *H19* can regulate downstream miRNAs (*miR-107*, *miR-27b*, *miR-106b*, *miR-125a,* and *miR-17*) during the process of osteogenic differentiation of MSCs to express ligands such as DII1, DII3, DII4, Jag1, and Jag2 (Liao et al. 2017). These ligands can regulate the Notch signaling pathway and promote BMP9 to induce osteogenic differentiation of MSCs, although the specific mechanism remains to be clarified. Studies have proved that activating the Notch signaling pathway through subcutaneous stem cell implantation and ectopic bone formation can restore the osteogenic deficiency of bone marrow mesenchymal stem cells caused by the knockdown of *H19*, and cell experiments in vitro were consistent with the results (Liao et al. 2017).

In addition, knocking down *H19* reduces the expression of SATB2 by up-regulating *miR-140-5p*. The *miR-140-5p* inhibits the osteogenic differentiation of BMSCs by targeting SATB2. Therefore, *H19* promotes the osteogenic differentiation of BMSCs by regulating the *miR-140-5p*/SATB2 axis (Bi et al. 2020).

Previous studies have shown that *miR-19b-3p* is a positive regulator of osteogenic differentiation (Xiong et al. 2020). The mimic transfection of *miR-19b-3p* can significantly promote the protein expression levels of RUNX2 and COL1A1, while the transfection of the *miR-19b-3p* inhibitor can significantly inhibit the expression of these two proteins. However, overexpression of *H19* can down-regulate the expression of *miR-19b-3p* in BMSCs. Therefore, *H19* is down-regulated in postmenopausal osteoporosis and promotes the up-regulation of *miR-19b-3p*. This controls the proliferation and osteogenic differentiation of BMSCs (Xiaoling et al. 2020).

### Roles of *H19* as ceRNA to regulate osteogenic differentiation

Recently, the ceRNA hypothesis proposed that lncRNA communicates with other protein-coding RNA transcripts by sharing a common miRNA binding site (Salmena et al. 2011). A large number of lncRNAs, including *H19*, are thought to play the role of a miRNA sponge (Liang et al. 2016). Through bioinformatics and genetic testing, Liang et al. found that *H19* is the miRNA sponge of *miR141* and *miR22*, both of which are negative regulators of osteogenesis and the Wnt/βcatenin pathway. *H19* competitively binds to the miRNAs and antagonizes the functions of the two miRNAs. Their target genes β-catenin aggregate, thereby activating the Wnt/β-catenin pathway to promote osteogenic differentiation (Liang et al. 2016).

*H19* can also act as the ceRNA of *miR138*, and then up-regulate the downstream FAK, and play a positive regulatory role in osteogenic differentiation. The specific mechanism involves *miR-138* inhibiting PTK2 gene expression through competitively binding with *H19* and promoting FAK expression to induce osteogenic differentiation of MSCs (Wu et al. 2018a, b).

**Fig. 2:**
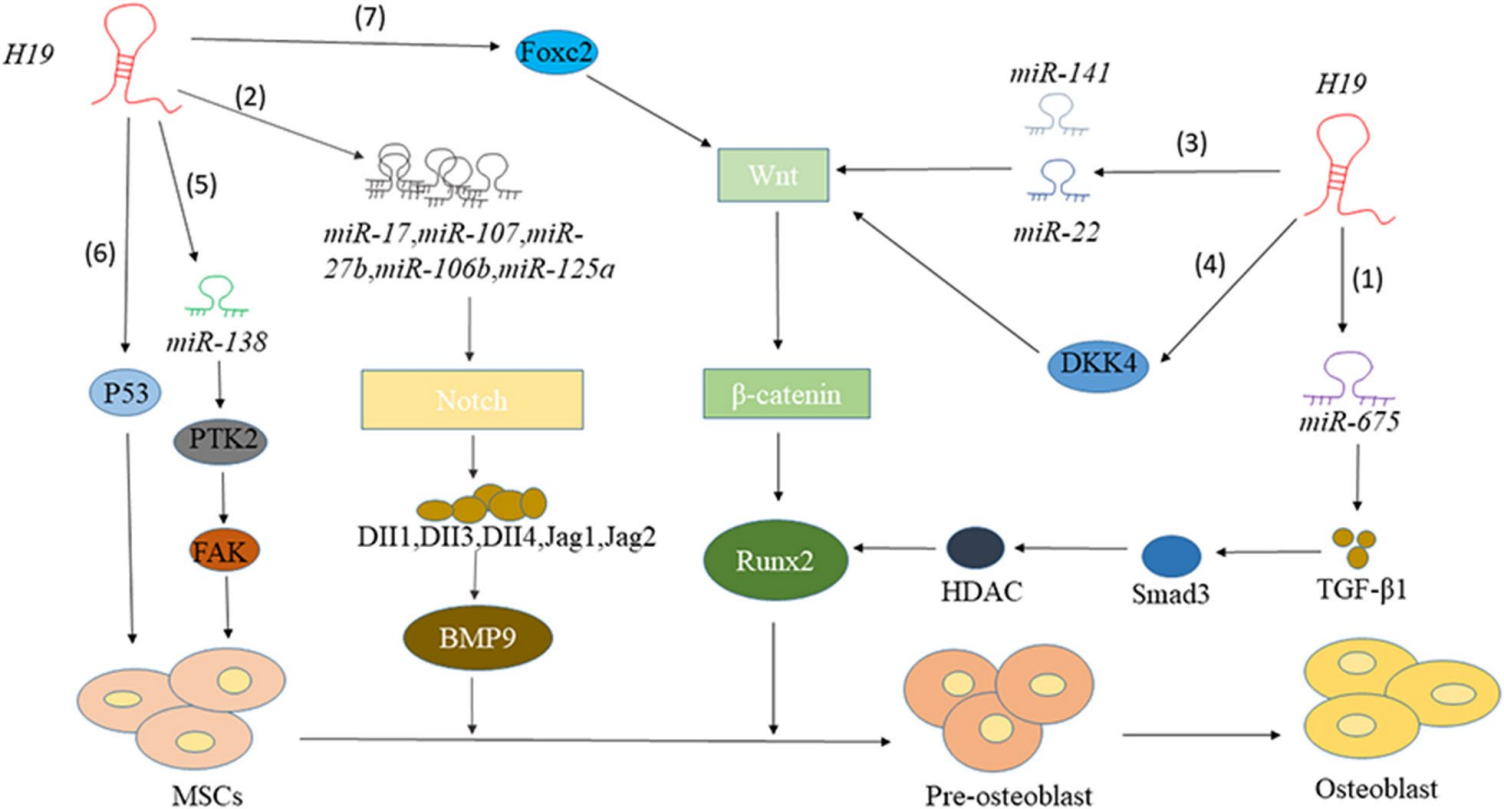
Mechanism of *H19* regulating osteogenic differentiation. (**1**) *H19* Promotes osteoblast differentiation via the TGF-β1/Smad3/HDAC signaling pathway by deriving *miR-675*. (**2**) *H19* regulates the Notch signaling pathway by regulating downstream miRNA expression ligands to promote the osteogenic differentiation of MSCs induced by BMP9 (**3**) *H19* is the miRNA sponge of *miR141* and *miR22*, which antagonizes the negative regulatory effects of two miRNAs on the Wnt/βcatenin pathway. (**4**) *H19* targets DKK4 to halt its inhibitory effect on the Wnt signaling pathway and osteogenic function. (**5**) *miR-138* sponge reduces the expression of PTK2 gene by competitively binding with *H19*, promotes FAK expression, and induces osteogenic differentiation of MSCs. (**6**) *H19* promotes the proliferation of osteoblasts by inhibiting the expression of p53. (**7**) The combination of *H19* and Foxc2 synergistically promotes the osteogenic differentiation of bone marrow mesenchymal stem cells

The *miR-188* is down-regulated in the osteogenic differentiation of mouse bone marrow mesenchymal stem cells (mBMSCs), while *H19* and LCoR are up-regulated in the adipogenic differentiation of mBMSCs (Wang et al. 2018). Knockdown of *H19* can significantly increase the expression of *miR-188*. Therefore, as shown in Fig. 2, H19 can mediate LCoR and regulate the balance between osteogenic and adipogenic differentiation of mBMSCs through sponging *miR-188*.

### Roles of *H19* in regulating factors related to osteogenic differentiation

*H19* also participates in osteogenic differentiation by regulating some factors associated with osteogenic differentiation (Table 1). For instance, Li et al. constructed a disused osteoporosis model after mechanically unloading mice and detected low expression of *H19* and high expression of DKK4 in these mice through gene sequencing (Li et al. 2017). The DKK family (DKK1-4) is an extracellular inhibitor of the Wnt/β-catenin pathway and plays a negative role in the process of osteogenesis (Aslan et al. 2006; Fujita and Janz 2007; Hiramitsu et al. 2013). Bioinformatics analysis showed that DKK4 is the target gene of *H19*, and through the Wnt/β-catenin pathway to mediate the biological effects of *H19* in disused osteoporosis (Li et al. 2017). Then, cell experiments were conducted to verify this, which resulted in the discovery that knockdown of *H19* can inhibit the Wnt/β-catenin pathway and osteogenic function. However, the knockdown of DKK4 can prevent this effect. Therefore, this result suggests that *H19* targets DKK4 to stimulate the Wnt/β-catenin pathway and promotes osteogenesis to prevent disused osteoporosis (Li et al. 2017).

**Table.1:** Different roles of H19 in promoting osteogenic differentiation

Expression of *H19*	Sampling tissue	Mode of action	Targets and pathways affected	Changes of targets	Refs.
Up-regulated	Human BMSCs	Precursor of *miR-675*	*miR-675*/TGF-β1/Smad3/HDAC axis Promoting Wnt/β-catenin pathway	Up-regulated of *miR-675*	Huang et al. (2015)
Up-regulated	Mouse BMSCs	Regulating miRNAs that target Notch ligands and receptors	miRNAs/ligands/Notch/BMP9 axis Promoting Notch pathway	Up-regulated of miRNAs	Liao et al. (2017)
Up-regulated	Human serum and Mouse BMSCs	Binding to FOX2	FOX2 Promoting Wnt/β-catenin pathway	Up-regulated	Zhou et al. (2019)
Up-regulated	Femurs of rats	Targeting DKK4	DKK4 Promoting Wnt/β-catenin pathway	Down-regulated	Li et al. (2017)
Down-regulated	Human blood and Human BMSCs	Mediating *miR-19b-3p*	*miR-19b-3p* *H19*/*miR-19b-3p*/RUNX2 axis	Up-regulated	Xiaoling et al. (2020)
Up-regulated	Tibia of mice	Binding to P53	P53	Down-regulated	Zhou et al. (2018)
Up-regulated	Human BMSCs	Sponging for *miR-138*	*miR-138*/FAK/ERK/Runx2 axis	Up-regulated	Wu et al. (2018a, b)
Up-regulated	Human BMSCs	Sponging for *miR-141* and *miR-22*	*miR-141* and *miR-22* Promoting Wnt/β-catenin pathway	Up-regulated	Liang et al. (2016)
Up-regulated	Mouse BMSCs	Sponging for *miR-188*	*H19*/*miR-188*/LCoR axis	Down-regulated	Wang et al. (2018)
Up-regulated	Human BMSCs	Sponging for *miR-140-5p*	*H19miR-140-5p*/SATB2 axis	Down-regulated of *miR-140-5p*	Bi et al. (2020)
Up-regulated	Human BMSCs	Sponging for *miR-214-5p*	*H19*/*miR-214-5p*/BMP2 axis	Down-regulated of *miR-214-5p*	He et al. (﻿^2021^)

*BMSCs* bone marrow mesenchymal cells, *miRNAs* microRNAs, *HADC* Histone deacetylase, *BMPs* bone morphogenetic proteins, *FOXC2* Forkhead box c2, *DKK4* Dickkopf protein 4, *FAK* Focal adhesion kinase, *ERK* extracellular signal-regulated kinase, *LCoR* Ligand-dependent corepressor, *SATB2* special AT-rich sequence-binding protein 2

Forkhead box c2 (Foxc2) is a member of the winged-helix/forkhead transcription factor family and participates in many biological processes. Overexpression of Foxc2 in bone marrow mesenchymal stem cells can promote osteogenic differentiation and inhibit adipogenic differentiation (You et al. 2013). At the same time, Foxc2 stimulates osteoblast differentiation of mesenchymal cells by activating the Wnt/β-catenin pathway (Kim et al. 2009). Foxc2 can promote the transcription of Wnt4 promoter by combining with the Wnt4 promoter, and the overexpression of Foxc2 in BMSCs can promote osteogenic differentiation through the Wnt/β-catenin pathway. The overexpression of *H19* can increase the expression of Wnt4, and the Wnt4 luciferase report shows that the activity of the Wnt-β/catenin pathway is enhanced after Foxc2 co-transfection with *H19* (Zhou et al. 2019). Therefore, the combination of *H19* and Foxc2 can promote the osteogenic differentiation of bone marrow mesenchymal stem cells through the Wnt/β-catenin pathway.

In addition to these pathways and regulatory mechanisms, research on how *H19* regulates osteogenic differentiation is gradually increasing. Zhou et al. established a tibial fracture model in mice to detect the expression levels of *H19* and p53 (Zhou et al. 2018). The experimental results show that in the process of osteogenic differentiation of MSCs, *H19* directly binds to p53 and inhibits the activity of p53 to promote the osteogenic differentiation of MSCs. Therefore, *H19*, as a molecular marker to promote fracture healing, induces the proliferation of bone cells by inhibiting the expression of p53.

### Roles of *H19* in regulating osteoclasts

The important role of lncRNA in osteoporosis has attracted the attention of researchers. However, there is little research on the role of *H19* in the proliferation and differentiation of osteoclasts. Some studies have shown that *miR-29a-3p* is the target of *H19,* and the expression of *H19* is up-regulated in patients with osteoporosis (Li et al. 2020). The overexpression of *H19* can promote the expression of inflammatory mediators such as TNF-α, IL-1β, and IL-10 by osteoclasts. After the knockdown of *H19*, the promotion effect is inhibited, and the proliferation of osteoclasts is also inhibited. Additionally, after co-transfection with the *miR-29a-3p* inhibitor and siRNA-*H19*, *H19* has a weakened regulatory effect on the expression of pro-inflammatory mediators, cell proliferation, and apoptosis. Therefore, the *H19*/*miR-29a-3p* axis is also involved in the development of osteoporosis by regulating osteoclasts (Li et al. 2020). Furthermore, there are some studies on *H19* in the blood of patients with osteoporosis. According to the latest research, *H19* is down-regulated in the plasma of postmenopausal patients with osteoporosis and can control osteogenic differentiation by targeting related factors to participate in osteoporosis. This is consistent with the results of previous studies (Xiaoling et al. 2020).

### DNA methylation of *H19* in the osteogenic differentiation

Currently, epigenetics is developing rapidly, and DNA methylation regulation is one of the key components being studied (Meng et al. 2015). Two genetic imprinting genes, IGF2/*H19*, are located on the 11p15 5 chromosomal regions. IGF2 is a maternally imprinted gene and is expressed by the father, while *H19* is paternal and expressed by the mother (Nordin et al. 2014). *H19* and IGF2 are expressed by opposite parental alleles but are jointly regulated. They share a common imprinting mechanism and are downregulated in many human cancers and fetal growth syndromes. A study found that when the methylation defect occurred in this region, it led to Beckwith–Wiedemann syndrome (BWS) (Fontana et al. 2021). The combination of *H19* and S-adenosylhomocysteine hydrolase (SAHH) can interfere with the hydrolysis ability of S-adenosylhomocysteine (SAH), and SAH is an effective product inhibitor of adenylate-dependent methyltransferase. This process leads to defective methylation of the targeted gene, which affects its activity (Zhou et al. 2015). In addition, the osteogenic activity in the aortic valve is controlled by NOTCH1, one of the members of the Notch family, which regulates the expression of key osteogenic genes, such as RUNX2 and BMP2. During calcific aortic valve disease, DNA methylation in the *H19* promoter is dysregulated, causing *H19* to interfere with the expression of NOTCH1 to promote the osteogenesis process in the aortic valve (Hadji et al. 2016). This evidence proves that *H19* can affect osteogenic differentiation through DNA methylation. The research on the relationship between the methylation status of gene imprinting and osteoporosis will be an emerging research direction in the field of bone metabolism. An in-depth understanding of the DNA methylation mechanism of *H19* will provide a new direction for the treatment of osteoporosis.

## Conclusion

In this review, we have been elaborating on the common functions of lncRNA, focusing on summarizing the role of *H19* in osteogenic differentiation and how it participates in osteoporotic activities. The roles of *H19* in osteogenic differentiation can be described in three ways: LncRNA *H19* directly participates in osteogenesis through the lncRNA–miRNA–mRNA network. For instance, *H19* promotes osteogenic differentiation through the *H19*/*miR-675*/TGF-β1/Smad3/HDAC pathway (Huang et al. 2015). Another role of *H19* is to indirectly participate in osteogenesis through the action of ceRNA, by absorbing miRNAs and competing with miRNA to regulate the encoded protein. Finally, lncRNA *H19* regulates osteogenic differentiation by influencing certain factors. In addition, studies have found that *H19* mediates estrogen-regulated osteogenic differentiation in BMSCs through the *miR-532-3p*/SIRT1 axis, and estrogen can mediate *miR-532-3p*/SIRT1 axis to reduce osteoporosis in ovariectomy rats by up-regulating *H19* (Li et al. 2021). These findings provide new ideas for the incidence and development of osteoporosis and provide new targets for its prevention and treatment. However, the regulation mechanism of *H19* has not been fully elucidated, and the effect of *H19* on downstream molecules is still unknown. The clinical application of *H19* and its use in the treatment of bone metabolism diseases are key topics for future studies.

## Data Availability

Not applicable.

## Abbreviations

lncRNAs: Long non-coding RNAs
miRNAs: MicroRNAs
ceRNAs: Competing endogenous RNAs
MSCs: Mesenchymal stem cells
BMSCs: Bone marrow mesenchymal stem cells
hBMSCs: Human Bone marrow mesenchymal stem cells
mBMSCs: Mouse Bone marrow mesenchymal stem cells
Bmncr: Bone marrow associated non-coding RNA
HDAC: Histone deacetylase
LCoR: Ligand-dependent corepressor
NF-κB: Nuclear factor-κB
MAPK: Mitogen-activated protein kinase
siRNA: Small interfering RNA
mRNA: Messenger RNA
ANCR: Anti-differentiation noncoding RNA
HOTAIR: HOX transcript antisense RNA
JNK: C-Jun amino terminal kinase
ERK: Extracellular signal-regulated kinase
SNHG1: Small nucleolar RNA host gene
RANK: Receptor activator of NF-kappaB
RANKL: Receptor activator of NF-kappaB ligand
CASC11: Cancer susceptibility 11
TNF: Transforming growth factor
GAS5: Growth arrest-specific transcript 5
HMGA1: High mobility group A1
DMNT1: DNA methyltransferase 1
DKK4: Dickkopf protein 4
Foxc2: Forkhead box c2
BMPs: Bone morphogenetic proteins
DII: Dietary inflammatory index
Jag: Jagged
SATB2: Special AT-rich sequence-binding protein 2
FAK: Focal adhesion kinase
PTK: Protein tyrosine kinases
SIRT1: Silencing information regulator 2 related enzyme 1
SAHH: S-adenosylhomocysteine hydrolase
SAH: S-adenosylhomocysteine
